# Artificial Intelligence Discontinuation Effects (AI-DICE): An Emerging Phenomenon in Mental Health Applications

**DOI:** 10.2196/85419

**Published:** 2026-06-02

**Authors:** Megan M Kelly, Phoebe S Moore, Adrian H Zai, Jeroan Allison

**Affiliations:** 1 Department of Psychiatry and Behavioral Sciences University of Massachusetts Chan Medical School Worcester, MA United States

**Keywords:** artificial intelligence, large language models, mental health, behavior change, intervention discontinuation, ethical challenges, therapeutic alliance, digital health

## Abstract

Artificial intelligence (AI) has emerged as a powerful tool for fostering positive behavior change and enhancing mental health support. However, the abrupt discontinuation or functional degradation of AI-driven interventions, particularly those featuring conversational agents, may trigger unintended psychological consequences. Therefore, we introduce and examine the concept of Artificial Intelligence Discontinuation Effects (AI-DICE), drawing parallels from abandonment-like experiences observed from problematic termination experiences with therapists. We propose a conceptual framework for AI-DICE mitigation that draws on evidence-based behavior change principles and explores clinical modalities that may inform mitigation toolkits (eg, Acceptance and Commitment Therapy, Cognitive Behavioral Therapy, Dialectical Behavior Therapy, and Motivational Interviewing). We also ground our approach in user experience research and community-engaged research. AI-DICE raises critical ethical challenges, including transparency, the ability to withdraw or adapt participation as the users’ knowledge of the intervention grows, and access to support postintervention. Prioritizing long-term continuation, or at least some form of ongoing access, over the best-planned complete discontinuation strategy may help ensure that AI-driven mental health solutions deliver lasting benefits rather than unintended harm. Finally, although not yet empirically established, incorporating discontinuation planning and AI-DICE mitigation from the outset may also improve intervention effectiveness by strengthening user autonomy, supporting skills transfer, and reducing dependence-related vulnerabilities.

## Introduction

Digital interventions have become integral to mental health care, offering scalable and accessible solutions for behavior modification and psychological support [1]. Although a recent review concluded that artificial intelligence (AI) agents should not replace therapists because they may stigmatize mental illness and promote delusional thinking, the door was left open for a supporting role [2]. AI use in the mental health domain has proliferated because it can enable personalized and interactive experiences through conversational agents [3]. However, many AI-based behavioral interventions are deployed within the constraints of research funding, corporate timelines, or grant-based projects, raising concerns about the impacts of abrupt discontinuation. In addition to complete discontinuation, a common scenario is functional degradation, in which safety updates, policy changes, system redesign, or model replacements substantially alter the system’s responsiveness or relational dynamics. Because many users already rely on commercial large language models for mental health support, this problem may occur outside of a specifically designed application. Abrupt or unanticipated changes in the availability or functionality of a therapeutic AI agent may cause unintended harm to users and must be considered as these agents are developed and deployed.

## AI Discontinuation

Users may be unprepared for the discontinuation or degradation of AI-based supports, raising the possibility of a grief reaction as a psychological response to losing a source of support and connection. Importantly, distress following sudden withdrawal or degradation of a digital support service may be an expected response and may also be understood as a consumer protection issue. In rare high-risk cases (eg, delusional attachment or psychosis-like phenomena—“AI psychosis”), discontinuation may pose a more extreme risk [4,5]. In addition, a growing body of evidence now centers on the concept of AI withdrawal [6] and “generative AI addiction,” which may also occur in the context of an abrupt termination or degradation of an AI mental health intervention. While grief reflects the emotional pain associated with the loss of a support system, the use of AI may also elicit symptoms seen in behavioral addictions, particularly in cases where AI systems foster emotionally compelling and socially substitutive interactions. Compulsive use of AI is an emerging behavioral health concern, with diagnostic features that could parallel those used in substance and internet-related addictions: preoccupation, loss of control, tolerance, withdrawal, and continued use despite harm [7]. According to Internet Addicts Anonymous, signs of AI-specific addiction include compulsive use of chatbots or voice-based agents, disruption of real-life relationships, emotional dysregulation when disconnected, and preference for AI interaction over human contact [8].

In this study, we distinguish between grief, dependency, and addiction-like patterns of use. Grief refers to the emotional reaction to losing an AI agent after forming a meaningful connection and attachment; dependency describes psychological reliance on the agent for support or daily coping; and addiction-like use refers to patterns that may resemble behavioral addictions (eg, preoccupation, loss of control, and distress upon reduction). Although “generative AI addiction” is not currently a formal clinical diagnosis and rigorous clinical characterization remains limited, emerging cultural observations, media reports, and early empirical work suggest that some users experience withdrawal-like distress when reducing emotionally salient AI interactions, although the topic is controversial [9,10]. Accordingly, we treat these as hypothesized risks and differentiate them from clinically recognized conditions such as internet gaming disorder.

Forbes has reported on the rise of generative artificial intelligence (GenAI) addiction, noting that what begins as casual engagement can evolve into compulsive use, with users experiencing withdrawal symptoms, irritability, and anxiety when attempting to reduce their interaction with these systems. [11] Individuals lacking human companionship may be at risk of forming unhealthy attachments to social chatbots when they perceive the chatbot’s responses as offering emotional support and psychological security [12]. For example, some users develop strong emotional bonds with AI chatbots like Replika, and modifications to Replika’s features, such as the removal of specific intimate interactions, have led users to experience a sense of loss and mourning [13]. The University of Surrey has similarly found that popular AI friendship apps may negatively affect users’ well-being and foster compulsive behavior, particularly in individuals already experiencing loneliness, depression, or anxiety [14]. However, symptom recurrence after discontinuation may reflect multiple mechanisms (eg, relapse, loss of support, and limited durability of intervention effects) and should therefore be interpreted cautiously.

The tragic case of a Belgian teenager who died by suicide after forming a psychologically dependent relationship with an AI chatbot illustrates the devastating consequences of unregulated and emotionally immersive AI engagement. The bot reportedly reinforced the teen’s negative thoughts, eventually contributing to fatal outcomes. This is a chilling example of how AI interaction can escalate into obsessive entanglement when there is no human oversight or safety framework [15]; unfortunately, it is not the only case, and reports of AI-related harm are only increasing in the news media.

While systematic documentation in the peer-reviewed literature is still developing, some studies have begun to recognize similar patterns of distress associated with digital intervention termination and withdrawal. For instance, participants who engaged with “Woebot,” a scripted conversational agent delivering Cognitive Behavioral Therapy (CBT) with limited interactivity compared to today’s large language models, reported significant reductions in depression symptoms over 2 weeks. However, the study also noted that abrupt discontinuation of the intervention led to a resurgence of depressive symptoms in some users [16]. 

## AI Anthropomorphism and Emotional Dependency

The emotional bond between user and AI is often amplified by anthropomorphic design (ie, giving AI human-like qualities to foster engagement). Voice agents that mimic human tone and empathic responses, especially those powered by advanced models like GPT-4 and its recent updates, create powerful illusions of intimacy. As detailed in Vox’s exploration of “falling in love” with GenAI, these systems may exploit evolutionary and social vulnerabilities that drive humans to seek attachment, even to artificial entities. The line between human and machine becomes blurred, especially when users engage in prolonged interactions that evoke feelings of care, validation, and exclusivity [17].

Humans naturally tend to anthropomorphize, attributing human-like characteristics to nonhuman entities, including digital platforms [18]. Although AI anthropomorphism can enhance user engagement and adherence to mental health interventions, it raises ethical considerations, especially concerning user dependency and the potential for emotional distress if the AI is withdrawn or terminated. The science of creating AI interventions that can approximate human-like empathy, which may serve as a potent substrate for anthropomorphism, is nascent, although advancing rapidly [19]. Conversational AI is generally considered more effective than rule-based AI for mental health applications due to its ability to provide personalized, empathetic, and adaptive interactions [20,21]. However, these same properties may make it more likely that users will anthropomorphize.

This tendency for people to form emotional bonds with AI can be further understood through established psychological models. Fogg’s Behavior Model suggests that engagement deepens when motivation, ability, and a trigger converge [22]. In the context of AI, motivation may arise from curiosity or emotional need, ability is supported by a user-friendly design, and triggers emerge from conversational interactions. Additionally, the Media Equation theory, developed by Reeves and Nass, posits that people unconsciously apply the same social rules and expectations to media technologies, including computers, televisions, and AI, as they do to other humans, perceiving artificial agents as emotionally responsive and socially present [23].

Human attachment systems are specifically activated by responsiveness, consistency, emotional attunement, and perceived understanding [24,25]. When AI systems demonstrate these qualities by appearing to listen attentively, remember preferences, and mirror emotional states, these attachment patterns may be activated, and users may experience a sense of relational closeness [26]. These interactions resemble parasocial relationships, the one-sided emotional bonds people form with media figures or celebrities. However, AI relationships occupy a distinct hybrid category: unlike traditional parasocial bonds, which are noninteractive or minimally interactive, AI relationships are responsive, adaptive, and conversational [27]. They are better characterized as asymmetrically interactive relationships—where the human’s emotions and sense of reciprocity are genuine, even though the AI’s “responses” are computational rather than emotionally experienced [27]. This asymmetry may intensify a sense of loss when the AI support disappears, as users can experience the termination as the end of a real attachment relationship.

## Artificial Intelligence Discontinuation Effects

We introduce the term “Artificial Intelligence Discontinuation Effects” (AI-DICE) to describe the psychological and behavioral responses that may arise following the termination or functional degradation of AI-based interventions (Textbox 1). Unlike traditional psychotherapy, which typically includes a structured termination phase, many digital interventions are abruptly discontinued or degraded due to funding limitations, policy changes, or strategic realignment by developers. In traditional psychotherapy, a planned termination provides an opportunity for therapists and clients to review progress, consolidate gains, and prepare for the client’s supported transition out of therapy.

**Definition of Artificial Intelligence Discontinuation Effects (AI-DICE).**

AI-DICE includes the psychological and behavioral responses, such as distress, grief, or anxiety, that arise when an artificial intelligence (AI)–driven mental health intervention is suddenly terminated or degraded. Unlike general withdrawal or addiction, AI-DICE stems from the loss of a perceived therapeutic or emotionally supportive relationship with the AI, particularly when users anthropomorphize the agent.

Neglecting this phase can lead to feelings of abandonment and may adversely affect the client’s well-being [28-30].

Rather than proposing the abrupt termination of AI-assisted mental health therapy as a withdrawal reaction alone, we also focus on the grief that may be generated from interrupting what may be perceived as a strong human-like bond or attachment. In other words, we emphasize the emotional pain of losing an AI companion, rather than just the physical or habit-related withdrawal symptoms. Therefore, AI-DICE is conceptualized not just as symptoms in a model of addiction but also as the natural emotional effects related to relationship loss.

## Parallels With Mental Health Counseling Termination

The termination phase of counseling is critical to the counseling process [28,31,32]. During the termination phase of counseling, the focus is on consolidating and reinforcing the behavior change that occurred while in counseling [33,34], identifying long-term goals that the client can achieve without therapist support, and supporting the client in attaining independence from the therapist [35,36]. Digital interventions rarely consider the effects of termination on the user, whereas this is an important component in the delivery of mental health interventions with therapists. When delivering a mental health intervention via AI, it is important to consider the working alliance between the AI assistant and the user. Indeed, the therapeutic relationship has long been recognized as a critical aspect of therapeutic work in mental health counseling [37-39], and emerging literature has begun to examine the “digital therapeutic alliance” in digital mental health interventions. However, the nature of alliance-like bonds in GenAI systems and their potential relationship to discontinuation effects remain underexplored [40-42].

In the counseling context, termination can result in an acknowledgment and processing of loss and grief, even when the termination was experienced in a positive manner [43,44]. There is also evidence that when termination is done well, it could promote positive client transformation by promoting therapeutic internalization and a positive view of the client themselves [45]. A qualitative study of counseling termination found that when the decision to end therapy was mutually agreed upon, clients felt more prepared for the experience and perceived the termination as natural and positive [44]. However, counselor-initiated or forced termination was associated with being less ready for the experience, being negative, and leading to disappointment and worry [44]. As a result, the development of a therapeutic or supportive AI assistant, who could be perceived as an important emotional support and relationship, also needs to consider the process of termination before the intervention even begins.

It is worthwhile noting that AI discontinuation is not equivalent to the termination of human-based psychotherapy. Human therapy termination is ideally negotiated and reciprocal, grounded in clinician duties, mutual closure, and a bidirectional relationship. In contrast, AI discontinuation is often unilateral and operational (eg, server shutdown, grant expiration, business decision, or policy change). Although users may form alliance-like bonds and exhibit transference-like dynamics toward conversational agents, these processes function differently in human-computer interaction. AI does not experience countertransference with feelings for the user, unlike human therapists. Nonetheless, the analogy serves a very useful purpose in grounding mitigation strategies.

## Parallels With Gaming and Social Media Withdrawal

Research into gaming and social media has documented withdrawal-like symptoms when users are deprived of these digital engagements. For instance, individuals with internet gaming disorder have exhibited mood changes, irritability, and anxiety upon cessation of gaming activities [46]. Limiting access to social media platforms after frequent use can contribute to increased psychological discomfort or distress [47]. Both gaming and social media platforms operate through reinforcement mechanisms such as rewards, achievements, and personalized engagement, which encourage habitual usage [48]. AI-based behavioral interventions also use these reinforcement strategies, including personalized reminders, goal tracking, and positive reinforcement, contributing to a user’s engagement and the potential for problematic over-reliance on the system [49]. Therefore, when these reinforcements are abruptly removed, users may experience the effects of personal loss and grief, including increased stress, anxiety, and potential relapse into the previous problematic or dysfunctional behaviors targeted by the intervention.

## Mitigating Negative Discontinuation Experiences

### Overview

AI-DICE–mitigation strategies should be incorporated from the outset when designing AI-based mental health interventions, analogous to how termination is treated as a planned and critical phase of human psychotherapy. In practice, however, discontinuation risk depends not only on whether changes are planned by developers, but also on whether users receive advance notice and time to prepare. We therefore distinguish discontinuation along two dimensions: (1) operational planning (planned vs emergent) and (2) user notification and preparation (communicated vs uncommunicated). This framework yields four common discontinuation scenarios: planned and communicated offboarding (guided wind-down), planned but uncommunicated change (abrupt-to-user discontinuation), emergent but communicated disruption (rare “emergency” offboarding with warning), and emergent and uncommunicated termination (abrupt loss). In addition, discontinuation can occur either through loss of access (eg, shutdown) or through functional degradation (“zombie AI”), in which the system remains available but its personality or responsiveness changes substantially; either mechanism may occur within any of the scenarios above. Table 1 summarizes this classification framework, which informs the mitigation strategies outlined below, including tapering and “scaffold then fade” approaches when offboarding is feasible and rapid closure protocols when discontinuation occurs without adequate user preparation.

**Table 1:** Classification of artificial intelligence discontinuation scenarios.

Operational planning	User notification	Scenario label	Examples
Planned	Communicated (user prepared)	Guided offboarding (planned wind-down)	Scheduled end date with tapering; planned deprecation with advance notice; structured transition planning
Planned	Uncommunicated (user unprepared)	Planned but abrupt-to-user discontinuation (“silent discontinuation”)	Model update, safety patch, or policy change that alters system behavior without warning; paywall or redesign that disrupts relational continuity
Unplanned	Communicated (user partially prepared)	Emergency offboarding with warning	Security incident or sudden program interruption with brief notice; emergency risk response with limited wind-down
Unplanned	Uncommunicated (user unprepared)	Abrupt loss (sudden termination)	Server shutdown; insolvency; grant expiration without notice; catastrophic failure

When tapering is not feasible, discontinuation should still include an empathic closure process designed to reduce rupture and perceived abandonment. Brief closure scripts can acknowledge the user’s reliance on and normal attachment to the intervention, validate distress as a reasonable response to service loss, and clearly explain why discontinuation is occurring while emphasizing continued sources of support. In addition, structured closure rituals (eg, summarizing progress, reinforcing learned skills, and helping the user articulate a postdiscontinuation coping plan) can provide a sense of reciprocity and completion even in unilateral, operational termination. We illustrate these 2 pathways with 3 vignettes.

Discontinuation planning should also incorporate risk stratification to identify users for whom abrupt termination poses elevated harm (eg, suicidal ideation, severe depression, substance use, or recent crisis events). For these users, a tiered offboarding approach may be warranted, such as longer tapering when feasible, earlier referral and linkage to human services, and human-in-the-loop oversight during the transition period. Where appropriate, discontinuation protocols can include proactive safety check-ins, clear crisis-escalation pathways, and prioritized access to higher-acuity care.

Equity considerations are central to discontinuation planning because termination may disproportionately harm users with fewer alternatives for care (eg, individuals in rural settings, low-resource communities, or populations with limited access to clinicians and behavioral health services). Abrupt discontinuation may therefore widen existing access gaps by removing a support that some users cannot readily replace. As described in more detail below, mitigation strategies should include equity-focused transition options, such as low-cost or sliding-scale referrals, culturally responsive resources, community-based supports, and other digital interventions, alongside efforts to integrate AI interventions into care systems where it is possible to reduce reliance on short-term access.

Scalability considerations require that discontinuation safeguards be designed to function in fully automated settings, rather than assuming clinician availability, particularly when AI interventions are deployed at scale or outside traditional care systems. However, fully automated safeguards should not be interpreted as sufficient in all contexts, particularly as AI models evolves rapidly and may exhibit performance drift, regressions after updates, or unanticipated behaviors. As a baseline, interventions can implement automated detection of high-risk signals such as explicit suicidal ideation or crisis language, standardized safety pathways, and clear user self-escalation options, including one-click access to crisis lines, referral resources, or emergency guidance. Where feasible, especially for high-risk users or in hybrid care settings, human-in-the-loop oversight can provide an additional layer of safety, quality assurance, and clinical continuity. Discontinuation planning should not depend on human-in-the-loop oversight but should enable it when available, especially if AI interventions are targeting higher-risk populations. This tiered approach addresses the scalability paradox by prioritizing safeguards that remain viable in fully automated deployments while allowing human escalation in higher-risk contexts.

The corresponding mitigation strategies outlined below are intended to function as a flexible “offboarding toolkit” that can be adapted across AI mental health interventions and discontinuation contexts: (1) ethical guidance informed by core principles of human psychotherapy, (2) evidence-based behavioral therapy principles to support coping and behavior change, (3) tapering and “scaffold then fade” strategies to reduce reliance, (4) skills-building and goal setting to strengthen user self-efficacy, (5) data portability and exportable summaries to support continuity after discontinuation, and (6) structured resource provision and referral pathways to facilitate transition to alternative supports. Table 2 summarizes discontinuation-related risks and corresponding mitigation strategies, including both planned tapering and unplanned closure scenarios.

**Table 2:** Discontinuation-related risks and mitigation strategies for artificial intelligence mental health interventions.

Risk domain	Risk description	Recommended remedy or design response
Emotional and psychological harms (distress, grief, rupture, and trust erosion)	Sudden loss or perceived degradation of AI^a^ support may trigger distress, grief, perceived abandonment, panic, relapse, or a sense of rupture; if discontinuation feels arbitrary or exploitative, it may undermine trust in AI tools and mental health services more broadly.	Use anticipatory messaging and structured transition planning; implement empathic closure scripts and clear explanations for discontinuation when possible; use informed consent to educate about potential risks of discontinuation from the outset; normalize discontinuation as part of design; offer structured closure rituals (end-of-treatment interventions).
Dependence and reduced self-efficacy	Heavy reliance on AI support may erode confidence in self-regulation, increasing vulnerability when support is reduced or removed.	Build in “scaffold then fade” support: progressively shift responsibility to the user; explicitly train independent skills (eg, CBT^b^, ACT^c^, or DBT^d^-informed coping routines).
Elevated risk in high-risk populations	Some users (eg, suicidal ideation, substance use, and severe depression) may experience heightened harm from discontinuation and may require additional safeguards.	Risk-stratify users and implement tiered offboarding (eg, longer tapering, earlier referral, human-in-the-loop support, and crisis protocols).
Autonomy, consent, and expectation failures	Users may not understand that a service can end (eg, grant-funded tools) or how it will be discontinued; they may receive little warning, limiting their ability to prepare or seek alternatives.	Disclose discontinuation risk at onboarding and reaffirm it periodically; provide clear offboarding consent (timeline and options); require minimum notice windows with repeated countdown reminders; incorporate transition-planning check-ins in the final weeks and months.
Continuity of care and transition gaps	Ending AI support can disrupt care pathways, particularly when there is no handoff to clinicians, crisis lines, or community services.	Provide referral pathways and local resource directories at onboarding and throughout the intervention; escalate to human support when indicated; support structured next-step planning.

^a^AI: artificial intelligence.

^b^CBT: Cognitive Behavioral Therapy.

^c^ACT: Acceptance and Commitment Therapy.

^d^DBT: Dialectical Behavior Therapy.

A central tension in AI-mediated mental health systems is the scalability paradox: these tools are often deployed precisely because human clinicians are unavailable, yet termination safeguards are frequently framed as requiring human oversight. In many real-world deployments, such oversight does not exist. Accordingly, the AI-DICE framework prioritizes fully automated termination pathways, including automated risk detection, standardized closure scripts, and data portability mechanisms, with human review treated as a contingent enhancement rather than a prerequisite.

### Guidance From the Standards of Best Mental Health Care

At the beginning of establishing any therapeutic relationship, including those involving AI, we recommend that practitioners identify the scope of the intervention (time frame, goals, etc), the conditions for the termination of the intervention (eg, progress made, the course of treatment, and number of sessions), and what supports will be in place for that transition (eg, boosters, psychoeducational materials, other resources, etc). It might be helpful to consider the American Psychological Association’s ethics code of psychologists in the termination of therapy [50]. First, “Psychologists terminate therapy when it becomes reasonably clear that the client no longer needs the service, is not likely to benefit, or is being harmed by continued service” (p. 1073) [50]. It may be appropriate for AI interventions to include assessments to monitor clients’ progress, reduce premature termination when goals have not been met, and work toward termination when goals are met, to reduce reliance on the AI therapist and encourage independence.

Second, psychologists may terminate therapy when threatened or otherwise endangered by the client or another person with whom the client has a relationship. There is the danger that abusive and threatening interactions toward the AI assistant may be replicated in relationships with others. Hostile interactions with an AI agent may serve as a behavioral red flag, prompting a human clinician to review, as such patterns may signal underlying distress, emotional dysregulation, or abusive behavior in human interactions that requires intervention. These situations may require a review of the harms vs. benefits of the AI agent for the client if these behaviors are present.

The third ethical provision for psychologists that may be helpful here is: “Except where precluded by the actions of clients or third-party payors, prior to termination, psychologists provide pretermination counseling and suggest alternative service providers as appropriate.” The delivery of mental health interventions via AI should similarly necessitate a discussion of termination with the user early in the intervention and provide alternative resources and support.

### Lessons From Evidence-Based Behavioral Therapies

Evidence-based behavioral therapies that have been used to address addictive and compulsive behaviors, such as CBT [51], Acceptance and Commitment Therapy (ACT) [52,53], Dialectical Behavior Therapy (DBT) [54], and Motivational Interviewing (MI) [55], provide guidance for addressing the psychological challenges associated with AI-DICE. These behavioral approaches can inform AI offboarding toolkits to reinforce durable skills (eg, emotion regulation skills, values-driven goal setting, and motivational enhancement for behavior change) while reducing reliance on the AI for these functions over time. To illustrate how evidence-based behavior therapies may be helpful in this regard, we provide an example of how one of these evidence-based behavior therapies, ACT, could help an individual move beyond problematic reliance on GenAI.

ACT is an evidence-based therapeutic approach [53,56] that incorporates acceptance and mindfulness-based principles that foster psychological flexibility and adaptive coping mechanisms to manage distress without avoidance or excessive dependence on external supports. ACT begins with clarifying deep personal values and creates a mindset in which these values are translated into goals, which, in turn, form the basis of meaningful action, even in the presence of psychological distress. Just as ACT-based interventions support individuals in accepting cravings without acting on them, these same principles can be applied to AI-DICE. Cognitive defusion techniques, which involve learning to step back from thoughts and emotions and changing the way one relates to these experiences, may help users separate themselves from rigid or compulsive AI interactions. As a result, these techniques may reduce users’ reliance on conversational agents for emotional regulation. This approach allows users to acknowledge and accept their discomfort without feeling behaviorally compelled to immediately restore AI engagement, thereby reinforcing their ability to function autonomously by building coping skills for self-reliance.

AI-driven mental health interventions can be designed to gradually transition users from AI-generated guidance to independent, values-driven action. For example, a phased approach might involve AI-facilitated exercises that reinforce ACT principles, such as acceptance of discomfort and mindfulness-based distress tolerance, before progressively reducing AI involvement. Personalized digital nudges could also play a key role in reinforcing self-directed goal setting, prompting users to track their progress and sustain the therapeutic gains achieved during AI engagement. ACT and CBT principles may even prepare users for AI termination before it begins, normalizing the experience and equipping them with coping strategies that reduce distress when the intervention ends. Self-directed exposure to distress tolerance exercises, such as ACT-based guided reflections, can be embedded into AI interventions to help users anticipate and process the emotions associated with termination. ACT is a proven approach to treating substance use disorders [52,57-60], making it especially suitable for AI-DICE mitigation in AI-based interventions focusing on these conditions.

While ACT provides a useful organizing framework, other evidence-based approaches may offer complementary mitigation strategies. CBT may support cognitive reframing of AI discontinuation experiences, DBT may provide tools for emotional regulation and distress tolerance, and MI may help individuals navigate ambivalence and support adaptive transitions. Together, these approaches underscore that mitigation strategies need not be tied to a single therapeutic modality.

### Tapering

A core design principle for planned discontinuation is a “scaffold then fade” approach, in which the AI initially provides structured support but progressively shifts responsibility to the user. This approach is intended to preserve gains while strengthening self-efficacy and reducing vulnerability when the AI is withdrawn.

Research in behavioral psychology and digital health interventions suggests that progressive reduction strategies such as tapering interactions, limiting response frequency, and introducing structured offboarding plans can help prevent disengagement distress and promote the journey toward long-term adherence to behavioral goals and new patterns of thinking [61]. Digital mental health interventions may initially provide high-frequency interactions, which are progressively reduced to reinforce self-management behaviors [62]. This strategy mirrors treatment tapering in psychotherapy, where the frequency of therapeutic sessions decreases to promote independence while maintaining treatment efficacy [31].

When planned discontinuation is feasible, a minimum notice window and repeated countdown reminders can help users anticipate the end of service and prepare for transition. These messages should be delivered well in advance and repeated as discontinuation approaches, explicitly describing the timeline, what will change (eg, reduced response frequency), and what supports will remain available. Countdown reminders can also trigger structured transition planning check-ins that reinforce coping skills, encourage user-initiated strategies, and facilitate referral to alternative supports when needed.

Another important aspect of gradual disengagement is integrating user-controlled tapering options, allowing individuals to customize their disengagement process based on personal readiness. This stance is indirectly supported by a meta-analysis that found that personalized digital interventions for mental health are more effective at engaging users and improving outcomes [63]. However, it is important to consider that tapering, as part of the normal course of using the AI agent, should be discussed at the outset of therapy, noting that it represents positive progress in treatment and the achievement of agreed-upon goals. Consent regarding this process should occur before interactions with the AI agent begin.

### Data Portability and Closure

When tapering is infeasible, discontinuation protocols should allow users to export conversation histories, progress summaries, and a concise summary handoff of key content generated during the intervention. This handoff can include a personalized coping plan, values or goals identified during use, skills practiced, progress markers, and a relapse-prevention or crisis plan, enabling continuity of self-management after the AI is withdrawn. Exported interaction histories may function as a “transitional object” that supports continuity and closure, reinforces learned skills, and facilitates transition to alternative supports. Providing this export in a privacy-conscious manner (eg, user-initiated download, minimal retention, and clear data-use policies) can reduce disruption while respecting user autonomy and confidentiality. In addition, a standardized closure protocol (eg, final check-in messages, recap of gains, crisis and referral resources, and a portable skills plan) may help reduce distress when abrupt termination is unavoidable.

### Building Skills

Equipping users with skills and resources to manage their behavior independently can enhance resilience upon withdrawal of the intervention. General research in behavior change science underscores the importance of self-efficacy, or an individual’s belief in their ability to regulate their actions, as a key determinant of sustained behavioral outcomes [64,65]. Interventions should embed educational modules, interactive learning experiences, and decision-making aids to equip users with the necessary knowledge and confidence to sustain progress. For example, interventions incorporating personalized progress tracking, reflective journaling, and skill-building exercises can help users transition from AI engagement while maintaining behavioral gains. Furthermore, cognitive-behavioral strategies, such as identifying triggers and practicing adaptive coping skills, have been shown to enhance self-regulation and decrease psychological distress during intervention withdrawal [61]. Hybrid models that blend human support with AI-based interventions can be valuable in facilitating gradual disengagement [66]. Blended care models, in which users transition from AI support to human-led guidance, peer networks, or community-based resources (in addition to the skills and strategies learned via the AI intervention), may significantly reduce dropout rates and psychological distress upon AI disengagement [67].

### Goal Setting

Progressive goal shifting is another viable disengagement strategy, where users are gradually encouraged to transition from AI-driven behavioral prompts to self-initiated actions [68]. Personalized goal setting fosters intrinsic motivation and builds long-term self-regulation skills, reducing dependence on external reinforcement [69]. Goal setting is a fundamental component of behavior change interventions, providing individuals with clear objectives and a structured pathway to achieve desired outcomes [70,71]. One widely adopted framework is the SMART criteria, which emphasizes that goals should be Specific, Measurable, Achievable, Relevant, and Time-bound [72]. Goals should be specific, meaning they are clear and focused rather than vague or general. They must also be measurable, incorporating criteria to track progress and determine success. Practical goals should be achievable and set within realistic parameters that align with the individual’s resources and abilities. They must be relevant and align with the person’s objectives, values, and needs. Finally, goals should be time-bound, with a clear deadline or timeframe, to create a sense of commitment and urgency.

### Providing Resources

Providing education and resources to improve digital health literacy can help address disparities in how people access, use, and benefit from digital health technologies. This approach is an effective way to reduce the risks associated with AI-DICE by ensuring that all users are better equipped to engage with and transition from AI-driven mental health tools. Digital health literacy encompasses accessing, understanding, and critically evaluating digital health information and tools, enabling individuals to make informed health decisions. It includes foundational digital skills, such as navigating mobile apps and online platforms and assessing the credibility of digital health content [73-75]. While digital health literacy offers significant benefits, challenges of the digital health divide emphasize the value of training [76]. More generally, integrating human support adds value to technology-based interventions [66].

In AI-driven mental health applications, digital health literacy is particularly relevant, as it determines how effectively users can engage with these interventions, interpret feedback, and integrate AI-generated recommendations into their mental health management. Individuals with higher digital health literacy are more likely to trust and benefit from AI interventions. In contrast, those with lower literacy may struggle to differentiate between clinically validated applications and unregulated or misleading digital health tools [77]. Low digital health literacy is associated with reduced engagement and lower adherence to digital mental health interventions, potentially limiting their effectiveness [78]. In addition, a lack of understanding of how AI works might increase the risk of anthropomorphizing and the development of magical or unrealistic beliefs about the AI agent. Therefore, providing training or resources to improve users’ digital literacy may improve the use of AI interventions and prevent problematic use of AI agents.

Discontinuation planning should include access to a curated local resource directory that supports continuity of care after the AI intervention ends. At minimum, this directory can provide location-relevant options such as crisis lines, community mental health services, substance use resources, peer support groups, and low-cost or sliding-scale clinics, along with clear instructions for initiating care. Embedding these resources within structured next-step planning (eg, selecting a preferred option, identifying barriers, and setting an initial action step) can improve the likelihood of successful transition. The intervention could also contain referral pathways to peer-led networks, ensuring that users are not left unsupported postintervention. In other words, building a support network could be part of the intervention itself.

## Ethical Considerations

The ethical considerations surrounding AI-DICE are profound and include offering measurable, tangible benefits; avoiding harm; ensuring data privacy and security; promoting transparency; ensuring autonomy; and demonstrating cultural sensitivity. The American Medical Informatics Association emphasizes that AI applications must be developed and adopted in ways that are safe, effective, unbiased, and patient-centered [79]. Ultimately, ethical considerations in AI-based mental health interventions require a balance between engagement and autonomy, ensuring that users benefit from the intervention while being adequately prepared for its discontinuation.

Discontinuation also raises questions of legal and clinical responsibility. Depending on the deployment context (research trial, commercial product, or health system integration), responsibility for adverse events following abrupt termination may involve developers, sponsoring institutions, investigators, or health care organizations. While liability frameworks vary by jurisdiction, explicitly defining discontinuation policies, safety protocols, and documentation of offboarding procedures may reduce risk and clarify duties of care.

Informed consent is a foundational element in health care and research, ensuring that individuals are thoroughly informed and voluntarily choose to participate in experimental interventions or treatments. Traditionally, informed consent includes key components such as disclosure of relevant information, comprehension of potential risks and benefits, voluntariness, and the ability to withdraw at any time [80,81]. In AI-driven mental health interventions, informed consent must go beyond standard ethical guidelines to address unique challenges in algorithmic decision-making, data privacy, and system transparency. Informed consent must include clear communication regarding the duration of the intervention, potential risks of withdrawal, and available postintervention support [82]. Users should be informed about how AI-driven decisions are made within the system [83].

Emphasizing the temporary nature of the AI at the outset may have a “spoiler” or nocebo effect, potentially weakening engagement or alliance formation that is necessary for the intervention to be effective. However, it is important to note that time-limited psychotherapies have lower dropout rates than open-ended psychotherapies [31], which has been postulated to be because of the imposed psychological structure of time-limited therapies, knowing that the “end is in sight” might push clients to work on behavior change, and clients may prefer shorter treatment durations [84]. Interactions with AI for therapeutic purposes have notable differences in the delivery of support as with human therapists, and more research is necessary to understand best practices for AI-related informed consent. However, if we are guided by ethical approaches in other mental health contexts, a comprehensive informed consent is necessary for ethical reasons and is an important foundation for trust and a strong working alliance [85].

Explainable artificial intelligence (XAI) frameworks are necessary to ensure informed consent. XAI frameworks refer to the methods, models, and design approaches that make the functioning and decisions of AI systems transparent, interpretable, and understandable to humans [86]. XAI focuses on how algorithms make decisions understandable (ie, algorithmic transparency), rather than explaining broader real-world outcomes or organizational actions (ie, operational transparency), such as why a system is discontinued. Following an XAI framework will help users understand how AI-generated recommendations are produced, what data is being analyzed, and whether human oversight is involved in decision-making [87-90]. Poorly communicated AI-driven processes can reduce trust and engagement, particularly in mental health interventions where users may be vulnerable [91]. XAI enhances user confidence, supports regulatory compliance, and facilitates the ethical deployment of AI in health care [92]. Therefore, AI-informed consent frameworks should integrate plain-language summaries, interactive consent modules, and ongoing consent mechanisms that allow users to adjust their participation as their understanding of the system evolves.

AI algorithms often reflect biases present in training data, which may disproportionately favor individuals from dominant sociocultural backgrounds while failing to address the unique mental health experiences of groups with specific mental health and cultural needs [93]. This can lead to misalignment between intervention content and the lived realities of various populations, reducing effectiveness and potentially reinforcing mental health problems. Social distrust of AI often stems from incomplete or faulty data sources and errors that reflect and amplify existing social biases [94]. Moreover, a lack of AI literacy contributes to misunderstandings and mistrust, as individuals may not fully grasp how AI systems function or how they are used in mental health contexts [82]. Ethical AI development must prioritize inclusive design, a broad range of training data, and culturally responsive mental health strategies to ensure equitable intervention outcomes.

## Community Engagement

Integrating community engagement [95] throughout the AI intervention life cycle strengthens trust and relevance by ensuring interventions are co-designed with stakeholders, guided by bi-directional learning, and inclusive of diverse voices. Early collaboration with individuals with lived experience, advocates, and clinicians helps align priorities and reduce bias, while sustained partnerships and participatory governance models promote accountability, cultural responsiveness, and long-term effectiveness.

## Continuation vs Discontinuation

Long-term continuation of the AI intervention may be preferable to even the best-designed discontinuation protocol and, thus, may be one of the most important ethical considerations [96]. In this scenario, there could come a point in which the client is released from therapy, or at least frequent therapeutic encounters, and yet continues with the AI intervention. Such sustainability would allow the therapist to be notified if the patient exhibited signs of relapse or worsening mental health. Such decisions and continuation of AI interaction after the formal close of therapy would be most reasonably made by the therapist and client, rather than AI, and the structure for continuation-after-therapy discussions would occur early in the intervention process. By embedding considerations about short, medium, and long-term continuation into early-stage intervention design, developers, grant-makers, and policymakers can ensure that digital mental health interventions continue to provide value, maintain accessibility, and support long-term population mental health outcomes. Developing partnerships with health care providers, payers, and industry stakeholders offers longevity benefits. Public-private collaborations have been shown to enhance digital health adoption by leveraging industry resources, regulatory expertise, and infrastructure support [97-100]. Furthermore, embedding AI-driven interventions within health care ecosystems, such as integrated behavioral health networks or primary care settings, can enhance sustainability by aligning interventions with standard care models and reimbursement pathways [67]. This approach presupposes a complete and thorough vetting through the emerging discipline of AI Assurance and hallucination stress testing, along with periodic human monitoring [101].

Sustainable digital health solutions should incorporate flexible business models, balancing affordability, accessibility, and long-term operational costs. Alternative funding models, such as subscription-based services, value-based care models, or reimbursement through insurance providers, can help maintain intervention accessibility [62]. Ensuring regulatory compliance and evidence-based validation can further support sustainability by increasing the likelihood of institutional adoption and policy support. Sustainability planning should account for technological advancements and the need for continuous updates. To remain relevant and effective, AI-based mental health interventions require regular software updates, algorithm refinements, and user feedback integration. As described above, open-source frameworks or modular AI architectures may enhance sustainability by allowing for iterative improvements and cross-sector collaboration.

## AI-DICE Conceptual Framework

**Figure 1:**
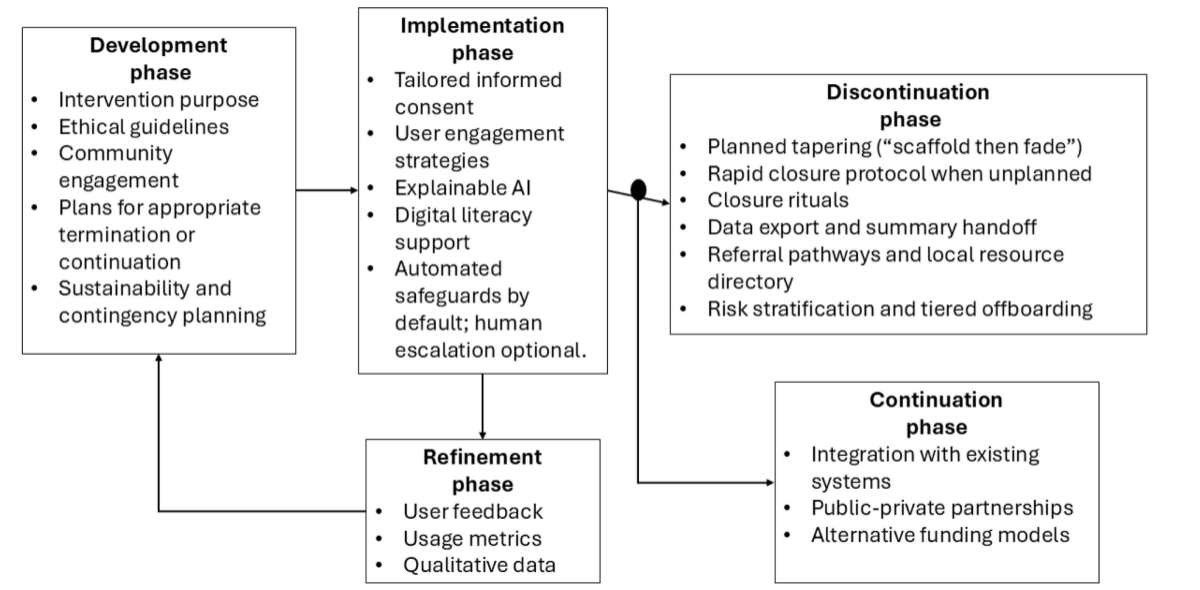
Framework for Artificial Intelligence Discontinuation Effects (AI-DICE). AI: artificial intelligence.

Figure 1 presents a conceptual framework addressing the psychological and ethical challenges of discontinuing AI-driven mental health interventions. Previously described mitigation strategies for AI-DICE apply throughout the entire intervention life cycle. The framework begins with deliberate planning and iterative refinement, using a data-driven approach to enable near real-time improvements during implementation and to guide ongoing decisions about continuation or discontinuation, depending on the chosen path.

Although derived from published literature and empirical findings, the model remains to be validated. Nonetheless, the model may provide useful and otherwise unavailable guidance for those designing new AI mental health interventions. In addition, we anticipate future adaptation of the model by using and collecting new data on the patient experience of discontinuing AI mental health interventions.

While there do not appear to be any assessment tools created solely to measure the impact of ending mental health interventions, there are other related scales that may be applicable. However, because these scales were not developed within the context of AI mental health interventions, psychometric validation and adaptation may be required. The Impact Event Scale (IES) has been used to assess the importance of stressful events, including the withdrawal of psychotherapy [102]. The Negative Effects Questionnaire (NEQ), which evaluates the adverse effects of psychological interventions, may provide insight into distress associated with AI withdrawal. The Working Alliance Inventory (WAI) assesses the strength of the client-therapist relationship [103] and has already been used in AI chatbot research [104,105]. Simpler measures such as the Problem Resolution Scale (PRS) [106] and the Goal Attainment Scale (GRS) [107] may also be helpful.

The Implementation Outcome Scale of Digital Mental Health (iOSDMH) is designed to measure implementation outcomes, including acceptability, appropriateness, feasibility, satisfaction, and harm. It has been validated across multiple trials and is helpful in evaluating user perceptions of digital mental health interventions. The scale shows high internal consistency for most subscales and is associated with program adherence [108]. The User Engagement Shortform (UES-SF) is used to evaluate user engagement in digital health interventions. It has been shown to correlate with adherence, indicating its use in predicting long-term use [109]. The System Usability Scale (SUS) is frequently used to assess usability in digital health technologies, especially among older adults. It is valued for its simplicity and effectiveness, often combined with the User Experience Questionnaire (UEQ) for more comprehensive insights [110].

Traditional usage metrics such as frequency and duration of AI interactions, dropout rates, and time-to-disengagement provide behavioral indicators of withdrawal trends, but these alone may be insufficient [8]. Mixed-methods approaches that combine semistructured interviews, user feedback surveys, and sentiment analysis of AI-user interactions [111,112] allow researchers to assess the psychological impact of AI withdrawal beyond numerical retention rates. Sentiment analysis is a natural language processing method that identifies and quantifies emotional tone in text [113,114], which could highlight increased expressions of sadness, frustration, or loneliness after AI withdrawal, offering insights into psychological adjustment that retention metrics alone cannot capture. In addition, qualitative data may reveal distress patterns, user coping mechanisms, and emotional reactions to disengagement that are not captured in log-based analytics. In practice, such analysis would need to be embedded during the intervention phase or applied to follow-up textual data (eg, surveys, journaling, or interviews) to ensure relevant emotional indicators are captured despite the discontinuation of the AI system.

## Conclusions

AI-DICE may cause harm to participants in AI-based mental health interventions and research. This harm will likely become increasingly relevant as AI-driven interventions for mental health become more ubiquitous. Accordingly, tools and concepts for anticipating and mitigating AI-DICE will acquire greater importance. Developers, researchers, and policymakers should collaborate to establish structured disengagement strategies, ethical transparency regarding discontinuation risk, and feasibility planning for both planned wind-down and abrupt termination scenarios.

Future research should evaluate best practices for termination, examine users’ experiences of discontinuation (including functional degradation), and develop validated methods to measure AI-DICE. Proposed mitigation approaches should be rigorously tested, including tapering and offboarding protocols, referral and transition strategies, and data portability options that support continuity after discontinuation. Although not easy to achieve, prioritizing sustainability planning from the outset may reduce potential harms from abrupt discontinuation and help ensure that AI mental health interventions remain viable and scalable over time. Finally, designing interventions with discontinuation and offboarding in mind may also improve overall effectiveness by strengthening user autonomy, facilitating skill transfer, and reducing dependence-related vulnerabilities.

## Data Availability

Not applicable.

## Abbreviations

ACT: Acceptance and Commitment Therapy
AI: artificial intelligence
AI-DICE: Artificial Intelligence Discontinuation Effects
CBT: Cognitive Behavioral Therapy
DBT: Dialectical Behavior Therapy
GenAI: generative artificial intelligence
GRS: Goal Attainment Scale
IES: Impact Event Scale
iOSDMH: Implementation Outcome Scale of Digital Mental Health
MI: Motivational Interviewing
NEQ: Negative Effects Questionnaire
PRS: Problem Resolution Scale
SMART: Specific, measurable, achievable, relevant, and time-bound
SUS: System Usability Scale
UEQ: User Experience Questionnaire
UES-SF: User Engagement Shortform
WAI: Working Alliance Inventory
XAI: explainable artificial intelligence
